# Molecular Surveillance of *Cronobacter* spp. Isolated from a Wide Variety of Foods from 44 Different Countries by Sequence Typing of 16S rRNA, *rpo*B and O-Antigen Genes

**DOI:** 10.3390/foods6050036

**Published:** 2017-05-11

**Authors:** Nancy Miranda, Pratik Banerjee, Steven Simpson, Khalil Kerdahi, Irshad M. Sulaiman

**Affiliations:** 1 Southeast Regional Laboratory, U.S. Food and Drug Administration, Atlanta, GA 30309, USA; Nancy.Vega@fda.hhs.gov (N.M.); Steven.Simpson@fda.hhs.gov (S.S.); Khalil.Kerdahi@fda.hhs.gov (K.K.); 2 Division of Epidemiology, Biostatistics, and Environmental Health, School of Public Health, University of Memphis, Memphis, TN 38152, USA; pbnerjee@memphis.edu

**Keywords:** *Cronobacter* spp., isolation, chromogenic and traditional media, molecular typing, foodborne disease

## Abstract

*Cronobacter* spp. are emerging infectious bacteria that can cause acute meningitis and necrotizing enterocolitis in neonatal and immunocompromised individuals. Although this opportunistic human-pathogenic microorganism has been isolated from a wide variety of food and environmental samples, it has been primarily linked to foodborne outbreaks associated with powdered infant formula. The U.S. Food and Drug Administration use the presence of these microbes as one of the criteria to assess food adulteration and to implement regulatory actions. In this study, we have examined 195 aliquots of enrichments from the nine major categories of foods (including baby and medical food, dairy products, dried food, frozen food, pet food, produce, ready-to-eat snacks, seafood, and spices) from 44 countries using conventional microbiological and molecular techniques. The typical colonies of *Cronobacter* were then identified by VITEK2 and real-time PCR. Subsequently, sequence typing was performed on the 51 recovered *Cronobacter* isolates at the 16S rRNA, *rpo*B and seven O-antigen loci for species identification in order to accomplish an effective surveillance program for the control and prevention of foodborne illnesses.

## 1. Introduction

*Cronobacter* spp. is a group of Gram-negative bacteria belonging to the family Enterobacteriacea which can survive in environments with extremely dry conditions. It is considered an emerging opportunistic pathogen capable of causing severe infections including necrotizing enterocolitis, bacteremia, and meningitis in humans [1,2,3]. In addition, this multi-species complex is typically facultative anaerobic, oxidase-negative, catalase-positive, rod-shaped, motile, non-spore forming bacteria that can predominantly produce a yellow pigment. Even though seven species of *Cronobacter* have been described (including *C. sakazakii*, *C. muytjensii*, *C. turicensis*, *C. dublinensis*, *C. malonaticus*, *C. universalis*, and *C. condimenti*), only three of these species (*C. turicensis*, *C. malonaticus*, and *C. Sakazakii*) have been associated with cases of infant death [4,5,6,7]. Importantly, a very wide temperature range (6 to −45 °C) has been reported for the typical growth of this group in brain heart infusion broth [4]. The growth of this group of organisms has also been recorded in powder infant formula (PIF) reconstituted at temperatures ranging from 8 to 47 °C [8]. It can survive for longer than two years in a desiccated state [3].

Although the natural reservoir of this organism is still unsettled, it has been isolated from a variety of food matrices that included produce, spices, herbs, and animal feed [9]. Nevertheless, the PIF has been associated with mostly neonate cases [10,11]. Furthermore, with the exception of *C. condimenti*, the rest of the six *Cronobacter* species have been associated with clinical infections; the *C. sakazakii* and *C. malonaticus* isolates have been reported to be primarily responsible for causing the majority of infant illnesses [6,7,12].

Thus far, DNA sequencing is considered the gold standard for rapid detection and species identification of human-pathogenic microorganisms of public health importance [13,14,15]. It has also been used to understand the population genetic structure, phylogenetic relationship, and taxonomic revision of various human-pathogenic bacteria causing foodborne illness. More recently, nucleotide sequence characterization is routinely employed in effective epidemiologic studies to reveal transmission routes of emerging infectious diseases, and in the prevention and control of various foodborne and waterborne diseases [13,14,15,16]. Multilocus sequence typing has been successfully used in the species identification of *Cronobacter* spp. worldwide [17].

Recently, we analyzed 195 food samples belonging to nine major food categories (including baby and medical food, dairy products, dried food, frozen food, pet food, produce, seafood, spices and ready-to-eat snacks), originated from 44 countries located on five continents (Americas, Africa, Asia, Europe, and Oceania) for the presence of *Salmonella*, *Listeria monocytogenes*, and *E. coli* in foods as part of an ongoing surveillance program for food safety of the agency (unpublished). In this study, we have tested the 195 aliquots of the above food enrichments for the presence of *Cronobacter*, and we have performed a two-step enrichment to aid the injured cells. Afterwards, the secondary enrichments were streaked on three chromogenic media and incubated at different temperatures to reduce the background flora and increase the odds of recovering the organism. Molecular typing was conducted on the recovered *Cronobacter* isolates for species identification by DNA sequencing of 16S rRNA, *rpo*B and seven sets of O-antigen loci.

## 2. Materials and Methods

### 2.1. Food Samples

This study examined a total of 195 aliquots of enrichments for the presence of *Cronobacter*. As listed in Table 1, the examined foods included baby and medical food (6 samples from 2 countries), dairy products (11 samples from 2 countries), dried food (7 samples from 5 countries), frozen food (5 samples from 3 countries), pet food (24 samples from 6 countries), produce (56 samples from 14 countries), seafood (11 samples from 9 countries), spices (54 samples from 21 countries), and ready-to-eat snacks (21 samples from 12 countries). The enrichments were initially made to isolate *Salmonella*, *Listeria monocytogenes*, and *E. coli* from the food samples in order to conduct a routine surveillance program for food safety of the agency, following the FDA Bacteriological Analytical Manual (BAM) [18].

### 2.2. Pre-Enrichment, Secondary Enrichment, and Culture on Chromogenic Media

In order to recover the *Cronobacter* spp. from the enrichment, aliquots of one mL of the refrigerated enrichment broth were aseptically added to 9 mL of pre-warmed BPW (at 43.5 °C in an incubator), and incubated at 37 °C for 16–24 h. After incubation, one mL portions of the enrichment were then transferred to 9 mL of R&F *Enterobacter sakazakii* enrichment broth (R & F Laboratories, Downers Grove, IL, USA) and 9 mL of Al-Holy-Rasco (AR) broth, and incubated at 43.5 ± 0.5 °C for 24 ± 2 h, as described above [19]. After completion of incubation, a loop full of R&F and AR enrichment broths were plated onto the Druggan-Forsythe-Iversen (DFI, Oxoid, Basingstoke, UK) and R&F *Enterobacter sakazakii* (*Cronobacter*) chromogenic agar plates by streaking at least three quadrants followed by incubation overnight, at 36 ± 1 °C for the DFI agar and 42 ± 1 °C for the R&F *E. sakazakii* agar respectively. After visualizing typical colonies on DFI and R&F agar plates, the colonies were transferred to *Enterobacter sakazakii* isolation agar (ESIA) (Oxoid, UK) and incubated at 43.5 ± 0.5 °C for 18–24 h following manufacturers’ recommendation for the reduction of background flora and better isolation of the target organism. The typical colonies isolated from ESIA were then transferred to Trypticase Soy Agar (TSA) with 5% Sheep Blood for identification and molecular analysis.

### 2.3. Isolate Identification

Identification of recovered isolates was achieved by making a bacterial suspension of purified culture raised on the TSA plates with 5% Sheep Blood. The colonies were suspended in 3 mL of sterile 0.45% saline, and its turbidity was verified and adjusted to achieve the necessary optical density for analysis using Biomérieux Vitek 2 System with the Vitek GN cards that range from 0.50–0.63 McFarland, following manufacturer’s instructions [20]. Once the run was completed, the isolates identified as the “*Cronobacter sakazakii* group” were transferred to BHI broth and incubated overnight at 37 ± 1 °C, for DNA extraction.

### 2.4. DNA Extraction and Real-Time PCR

The DNA extraction was achieved by using QIAGEN DNeasy Blood and Tissue kit following manufacturer’s protocol for the purification of total DNA from Gram-negative bacteria (QIAGEN, Valencia, CA, USA) for all of the food samples that tested positive for *Cronobacter* in the study (Table 2). For each isolate, the cell pellet from one milliliter bacterial culture grown overnight at 37 °C in BHI broth was used as the starting material. The concentration of purified genomic DNA was measured at 260 nm absorbance using a NanoDrop-1000 spectrophotometer (NanoDrop Technology, Rockland, DE, USA), and stored at −20 °C until used.

To perform the real time PCR analysis, two microliters of sample templates were examined following the FDA BAM Method without internal and using the Cepheid SmartCycler Thermal Cycler (software version 2.0 d, Cepheid, Sunnyvale, CA, USA) [18].

### 2.5. PCR Amplification

**Figure 1:**
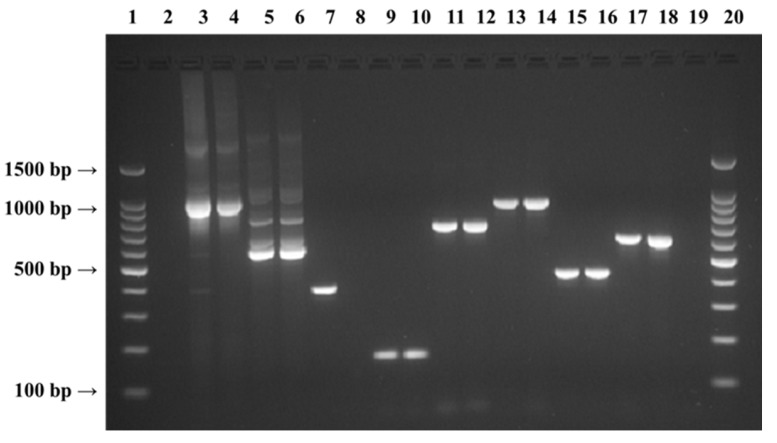
Agarose gel showing *Cronobacter*-specific PCR amplified products at eight different loci. Lane 1 and 20: Promega™ 100 bp DNA ladder molecular weight marker, Lane 3–4: 16S rRNA, Lane 5–6: *rpo*B, Lane 7: O-antigen 1, Lane 9–10: O-antigen 2, Lane 11–12: O-antigen 3, Lane 13–14: O-antigen 4, Lane 15–16: O-antigen 6, Lane 17–18: O-antigen 7, and Lane 2–8–19: Negative Control.

To amplify the regions of 16S rRNA, *rpo*B and the seven known *C. sakazakii*-specific O-antigen genes, nine unique PCR protocols were developed using the published PCR primer sets (Table 3). For 16S rRNA PCR amplification, a total of 50 µL PCR reaction consisted of 25 µL of HotStarTaq Master Mix (QIAGEN, this premixed solution contains HotStarTaq DNA Polymerase, PCR Buffer, and dNTPs with a final concentration of 1.5 mM MgCl_2_ and 200 µM each dNTP), and 25 µL of a solution containing 200 nM of each primer, 1.5 mM of additional MgCl_2_ (Promega, Madison, WI, USA) and template DNA (50 ng) diluted in PCR grade water. The PCR reactions were run for 35 cycles (each cycle is 94 °C for 45 s, 60 °C for 45 s, and 72 °C for 60 s) in a GeneAmp PCR 9700 thermocycler (Applied Biosystems, Foster City, CA, USA), with an initial hot start (94 °C for 15 min) and a final extension (72 °C for 10 min). The PCR conditions for *rpo*B and O-antigen amplification were similar to 16S rRNA except that the annealing temperature was 56 °C for *rpo*B and it was 50 °C for the O-antigen PCR amplification. The PCR products were examined by agarose gel electrophoresis and visualized after ethidium bromide staining (Figure 1).

### 2.6. DNA Sequencing and Data Analysis

In order to perform DNA sequencing, the amplified PCR products were enzymatically cleaned before cycle sequencing, 3 μL of ExoSAP-IT (USB Corporation, Cleveland, OH, USA) was added to 5 μL of each amplified PCR product, as described above [13]. The mixture was incubated at 37 °C for 20 min followed by 80 °C for 15 min on a GeneAmp PCR 9700 thermocycler (Applied Biosystems, Foster City, CA, USA). The purified PCR products were sequenced using AB Big-Dye 3.1 dye chemistry and AB 3500 XL automated DNA sequencers (Applied Biosystems) with sequencing reaction competed for 25 cycles (each cycle is 96 °C for 30 s, 50 °C for 15 s, and 60 °C for 4 min) and held at 4 °C in a GeneAmp PCR 9700 Thermocycler (Applied Biosystems). The cycle sequencing reactions contained 2 μL of cleaned PCR product, 1 μL of BigDye Terminator v3.1 Ready Reaction Mix, 2 μL of 5× Sequencing Buffer, 1.6 pmol of Forward or Reverse sequencing primer, and water in a final volume of 20 μL. Sequencing reactions were cleaned up with the Performa^®^ DTR Gel Filtration Cartridges following manufacturer’s protocol (Edge Bio, Gaithersburg, MD, USA). Sequence accuracy was confirmed by performing two-directional sequencing. Multiple alignments of the generated nucleotide sequences were carried out by using the BioEdit and Geneious programs with manual adjustments.

### 2.7. Nucleotide Sequence Accession Numbers

The generated nucleotide sequences of 16S rRNA, *rpo*B and the seven known *C. sakazakii*-specific O-antigen genes of the recovered *Cronobacter* spp. isolates were deposited in the GenBank database under accession numbers KY652858 to KY652894.

## 3. Results

In this surveillance study, a total of 195 enrichments of food samples belonging to nine recognized food categories ( I. baby and medical food; II. dairy products; III. dried food; IV. frozen food; V. pet food; VI. produce; VII. seafood; VIII. spices; and IX. ready-to-eat snacks) were examined initially following conventional microbiologic protocols for the presence of *Cronobacter* (Table 1). In addition, all 195 food samples were initially tested for the presence of *Salmonella*, *Listeria monocytogenes*, and *E. coli* as part of an ongoing surveillance program for food safety of the agency. There were 14 food samples that tested positive for these three bacterial species known to cause foodborne diseases; *Salmonella* was detected in half of the 14 food samples tested (data not shown).

Of the various foods examined for *Cronobacter*, the *Cronobacter*-specific typical colonies were observed for 51 of the food samples tested (Table 2). The initial biochemical screening for the recovered *Cronobacter* isolates from typical colonies was achieved by using Biomérieux Vitek 2 System and *Cronobacter*-specific QPCR (Table 2). All food samples belonging to the baby and medical food and dairy product categories were found to be negative for the presence of *Cronobacter*. Nevertheless, some of the samples from the rest of the seven food categories were positive for the presence of *Cronobacter* that included 28.6% of the dried foods, 20.0% of the frozen food, 29.1% of the pet food, 17.8% of the produce, 9.1% of the seafood, 44.4% of the spices, and 23.8% of the ready-to-eat snacks, food samples investigated (data not shown). Afterward, PCR was performed on these recovered *Cronobacter* isolates targeting the 16S rRNA. *rpo*B, and the seven *Cronobacter sakazakii* O-antigen (O1 to O7) genes for species identification. Sequence characterization was carried out on the PCR amplified products of seven *Cronobacter sakazakii* O-antigen (O1 to O7) loci for the identification of *Cronobacter sakazakii* O serotypes (Table 4). DNA sequencing of PCR-amplified products of 16S rRNA and *rpo*B genes were also performed for nine isolates (Table 4), which were found to be QPCR positive but PCR-negative for all of the seven O-antigen serotypes primers tested. In all cases, the published primer sets that are listed in Table 3 were tested against the genomic DNA of the specimen with modified PCR conditions at least three times using the HotStarTaq Master Mix kit (QIAGEN), for their sensitivity and robustness by completing PCR amplification. The bi-directional nucleotide sequencing was done on the PCR amplified products for each of the three genes examined. The previously described generic primer sets based on the conserved regions of rRNA [21] and *rpo*B [22] loci known to provide genotypic bacterial identification, resulted in PCR products of approximately 1000 bp and 550 bp in size, for the 16S rRNA and *rpo*B regions amplified, respectively (Figure 1). Furthermore, in this study, the published *Cronobacter sakazakii* O-antigen (O1 to O7) gene specific primer sets [23] generated the PCR amplified products for six of the seven O-antigen serotypes tested: serotype O1, 364 bp; serotype O2,152 bp; serotype O3,704 bp; serotype O4, 890 bp; serotype O6, 424 bp; and serotype O7, 615 bp (Figure 1).

All of the recovered *Cronobacter* isolates from the 51 food samples were amplified at the 16S rRNA and the *rpo*B loci (Table 2). Nucleotide sequencing of 16S rRNA and the *rpo*B revealed a considerable inter- as well as intra- specific genetic variation among the recovered *Cronobacter* isolates characterized, and apparently the 16S rRNA regions displayed less polymorphism as compared to the *rpo*B gene (Table 4).

Of the 51 recovered *Cronobacter* isolates from the 195 different foods, none of the isolates were found positive for O-antigen 5 serotype; distinct *Cronobacter sakazakii* O serotypes were identified among the 51 recovered isolates from various foods (Table 2). However, a significant genetic polymorphism was observed at the rest of the six O-antigen loci sequenced; distinct *Cronobacter sakazakii* O serotypes were identified among the 51 recovered isolates from various foods (Table 1). Three unique sequence patterns were noticed among the *Cronobacter sakazakii* O serotype 1 isolates; no genetic polymorphisms was observed among the recovered *Cronobacter sakazakii* O serotype 2, serotype 3, and serotype 4 isolates which matched 100% with respective published sequence available in GenBank (Table 2). The *Cronobacter sakazakii* O serotype 6 and serotype 7 also displayed unique sequence patterns; some of the sequences matched 100% with the published sequences and showed minor genetic variation (Table 4).

## 4. Discussion

The primary mission of FDA, being a regulatory agency, is to forbid distribution of hazardous Food, Drug and Cosmetic products, and to keep their supply chain safe. Recently, the remarkable increase in the international production of FDA-regulated commodities (including ingredients and finished products) has made it very challenging to accomplish this mission. This agency uses the presence of human-pathogenic *Cronobacter* spp. as one of the criteria in implementing regulatory actions and assessing adulteration of foods.

The *Cronobacter* spp. has been linked primarily to a number of foodborne outbreaks associated with PIF contaminations, and even a lower dose of infection by this pathogen can be life-threatening in neonates [24,25]. Since the discovery of *Cronobacter*, several conventional culture methods have been described for the isolation of *Cronobacter* spp. [26]. The use of chromogenic selective media with a real-time PCR based confirmatory molecular test was considered to be advantageous for rapid screening and identification of *Cronobacter* species [27]. In a recent study, it was suggested that incubation at 30 °C may be suitable for the recovery of some *Cronobacter* species and minor variations in growth conditions can alter colony morphology and appearance. This may also promote the expression of unique biological characteristics based on phenotypic observations which may be beneficial for differentiating various *Cronobacter* strains [28].

To date, the ribosomal RNA (rRNA) is considered to be the most conserved region of genomes having several copies and a slower rate of evolution. Therefore, it has been most extensively sequenced to understand the genetic diversity across the prokaryotes and eukaryotes. It has also been most widely used as a phylogenetic marker to understand taxonomic and evolutionary relationships and for the development of molecular diagnostic methods [3,15,29,30]. The 16S rRNA gene based PCR identification system was reported to be a specific and reliable tool that could correctly identify *C. sakazakii* isolates from distinct phylogenetic lines [21]. Later, the *rpo*B gene was described as an effective genetic marker for bacterial identification and phylogeny; a *rpo*B based PCR systems was developed and evaluated to differentiate the six proposed species within the *Cronobacter* genus [22,31]. Further, variation in the O-antigen lipopolysaccharide was considered and utilized for serotyping the Gram-negative bacteria. The O-antigen serotyping scheme for *C. sakazakii* (that includes seven serotypes O1 to O7) was recently recognized, and the O-antigen gene clusters and specific primers were developed for the identification of *C. sakazakii* O1 to O7 strains [23,32]. The sensitivity of PCR assay was described by analyzing the serial dilutions of *C. sakazakii* O1 to O7 genomic DNA (10, 1, 0.1, 0.01, 0.001, 0.0001, and 0.00001 ng) and using it as a template. The sensitivity of the *C. sakazakii* O1 to O7 isolates in pure culture was also tested by culturing in Luria-Bertani (LB) medium to log phase followed by 10-fold serial dilution, and the CFU were counted after overnight incubation at 37 °C. [23]. The seven sets of O-antigen primers were further tested for their sensitivity and specificity by amplifying 136 *C. sakazakii* O1 to O7 isolates, isolates from other *Cronobacter* species (including two isolates each of *C. malonaticus*, *C. dublinensis* and *C. turicensis* strains, one isolate of *C. muytjensii*), and 10 isolates of closely related species which also included cross-testing of each primer sets with other O serotype isolates as well as with the closely related isolates [23]. Results based on the serial dilution (10 to 10^8^ CFU/mL) of pure cultures of *C. sakazakii* O serotypes O1 to O7 and using it as templates, revealed positive signals for all seven serotypes at 10^3^ CFU/mL dilution [23].

Furthermore, multilocus sequence typing (MLST) has been reported suitable for finding genetic polymorphism in microbes with low natural genetic diversity [33,34,35]. A 7-loci based *Cronobacter*-specific MLST was developed [36], and the MLST was subsequently used to understand genetic diversity of recovered *C. sakazakii* isolates from PIF, ingredients of PIF, and their production premises [37,38,39,40,41]. More recently, MLST was employed in the genetic characterization of *Cronobacter sakazakii* recovered from the environmental surveillance samples during a sporadic case investigation of foodborne illness [17].

In recent years, a number of surveillance studies have been carried out for the identification of *Cronobacter* spp. from foods, using conventional microbiological and molecular techniques worldwide. These surveillance studies include research on raw dried pasta from the German market [42], wheat flour from China [43], dehydrated rice powder from the Chinese Supermarket [44], dried food from Japan [45], retails foods from Brazil and Czech Republic [11,46], ready-to-eat foods other than infant formula from Ireland and Switzerland [47], ready-to-eat foods from China [48], herbs and spices from Jordon [49], medicinal plants, herbs, and spices from India [50], spices and herbs from Poland [51], infant formula production factory premises and powdered infant formula from China [52]. The molecular tools were also successfully used in the reevaluation of a suspected *Cronobacter sakazakii* outbreak in Mexico [53].

## 5. Conclusions

The 16S rRNA, *rpo*B and the seven *Cronobacter sakazakii*-specific O-antigen primer sets can be used for rapid detection and differentiation of *Cronobacter* spp. isolates recovered from the surveillance food samples. DNA sequencing of O-antigen 1–7 serotyping is an ideal tool for the genetic typing of *C. sakazakii* recovered isolates from foods. These unique molecular diagnostic tools can help the FDA accomplish its important mandate of the food safety program, and conduct epidemiologic surveillance and investigations of public health importance.

## Figures and Tables

**Table 1:** Food samples analyzed in the study with their countries of origin.

Origin (Region/Country)	Food Products Tested
**Americas**	
Argentina	chia seed, pet food
Belize	pet food, papaya
Brazil	papaya
Canada	pet food, sesame seed
Chile	chili powder
Colombia	basil
Costa Rica	papaya, coriander
Dominican Republic	cantaloupe, cilantro, cucumber, papaya
Ecuador	ready-to-eat snack
El Salvador	okra, spice powder
Guatemala	breading flour, mango, papaya, seasoned flour
Guyana	brown sauce
Haiti	mango
Honduras	cucumber
Jamaica	spice powder
Mexico	avocado, basil, cilantro, kale, octopus, yellow croaker
Nicaragua	cheese
Peru	paprika powder, shrimp
USA	alfalfa sprout, avocado, broccoli sprout, broccoli sprout seed, cheese, clover seed, cucumber, frozen ravioli, kale, organic clover sprout, parsley, pet food, powder infant formula, powder milk, ready-to-eat snack, spice powder, spinach, tomato
**Africa**	
Sao Vicente (Cape Verde)	ready-to-eat snack
Ghana	ogbono seed, smoked tilapia, spice
Kenya	spice powder
Morocco	ready-to-eat snack
South Africa	pepper, spice powder
**Asia**	
China	cauliflower, frozen crab cake, garlic powder, pet food, spice powder, tilapia
India	black pepper, crushed red pepper, garlic powder, sesame seed, spice powder, ready-to-eat snack, wafers wheels
Indonesia	spice powder, tilapia
Israel	basil
Malaysia	shrimp
Pakistan	ready-to-eat snack, spice powder
Philippines	cassava leaf, desiccated coconut
Sri Lanka	cinnamon, cinnamon quill
South Korea	ready-to-eat snack
Taiwan	black sesame powder, spice salt, spice powder
Thailand	ready-to-eat snack, spice powder
Turkey	laurel leaves, strawberry
Vietnam	ground black pepper, ready-to-eat snack, shrimp, tuna, white pepper
**Europe**	
Germany	chocolate powder, spice powder
Ireland	pet food, ready-to-eat snack
Italy	frozen linguine, ready-to-eat snack
Netherland	parsley leaf
Spain	cantaloupe, crawfish, paprika
UK	medical food, salmon
**Oceania**	
Australia	alfalfa beans

**Table 2:** Food samples that tested positive for *Cronobacter* in the study.

S. No.	Sample Number	Description of Food Products	Country of Origin	Food Product Type/Category	Bacterial Culture	QPCR	PCR Screening			* O-antigen Reference (Sequence Variation, % Similarity)	* O-antigen Sequence Type
							16S rRNA	*rpo*B	* O-antigen		
1	SRL-66	Cassava leaf	Philippines	Dried food	TG	+	+	+	6	JQ674749, This report (Identical)	*C. sakazakii*
2	SRL-80	Breading flour	Guatemala	Dried food	TG	+	+	+	1	CP011047, This report (Identical)	*C. sakazakii*
3	SRL-154	Frozen Ravioli	USA	Frozen food	TG	+	+	+	6	JQ674749, This report (4-point-mutation)	*C. sakazakii*
4	SRL-86	Pet food	USA	Pet food	TG	+	+	+	2	EU076546, This report (Identical)	*C. sakazakii*
5	SRL-91	Pet food	Canada	Pet food	TG	+	+	+	NOA	NOA	NOA
6	SRL-93	Pet food	Canada	Pet food	TG	+	+	+	1	CP000783, This report (Identical)	*C. sakazakii*
7	SRL-101	Pet food	Canada	Pet food	TG	+	+	+	1	CP000783, This report (Identical)	*C. sakazakii*
8	SRL-163	Pet food	China	Pet food	TG	+	+	+	2	EU076546, This report (Identical)	*C. sakazakii*
9	SRL-186	Pet food	USA	Pet food	TG	+	+	+	NOA	NOA	NOA
10	SRL-199	Pet food	China	Pet food	TG	+	+	+	1	CP011047, This report (Identical)	*C. sakazakii*
11	SRL-12	Basil	Mexico	Produce	TG	+	+	+	NOA	NOA	NOA
12	SRL-13	Parsley	USA	Produce	TG	+	+	+	NOA	NOA	NOA
13	SRL-20	Basil	Colombia	Produce	TG	+	+	+	1	CP000783, This report (1-point-mutation)	*C. sakazakii*
14	SRL-35	Alfalfa beans	Australia	Produce	TG	+	+	+	1	CP011047, This report (Identical)	*C. sakazakii*
15	SRL-42	Basil	Colombia	Produce	TG	+	+	+	NOA	NOA	NOA
16	SRL-87	Parsley leaf	Netherland	Produce	TG	+	+	+	2	EU076546, This report (Identical)	*C. sakazakii*
17	SRL-94	Alfalfa sprout	USA	Produce	TG	+	+	+	NOA	NOA	NOA
18	SRL-140	Avocado	USA	Produce	TG	+	+	+	6	JQ674749, This report (Identical)	*C. sakazakii*
19	SRL-173	Avocado	USA	Produce	TG	+	+	+	3	HQ646169, This report (Identical)	*C. sakazakii*
20	SRL-194	Avocado	USA	Produce	TG	+	+	+	6	JQ674749, This report (4-point-mutation)	*C. sakazakii*
21	SRL-95	Smoked Tilapia	Ghana	Seafood	TG	+	+	+	7	JQ674750, This report (2-point-mutation)	*C. sakazakii*
22	SRL-4	Garlic powder	India	Spice	TG	+	+	+	2	EU076546, This report (Identical)	*C. sakazakii*
23	SRL-36	Spice powder	Pakistan	Spice	TG	+	+	+	3	HQ646169, This report (Identical)	*C. sakazakii*
24	SRL-40	Spice powder	South Africa	Spice	TG	+	+	+	2	EU076546, This report (Identical)	*C. sakazakii*
25	SRL-41	Pepper	South Africa	Spice	TG	+	+	+	1	CP011047, This report (Identical)	*C. sakazakii*
26	SRL-43	Spice salt	Taiwan	Spice	TG	+	+	+	2	EU076546, This report (Identical)	*C. sakazakii*
27	SRL-47	Black sesame powder	Taiwan	Spice	TG	+	+	+	2	EU076546, This report (Identical)	*C. sakazakii*
28	SRL-56	Spice powder	India	Spice	TG	+	+	+	1	CP011047, This report (Identical)	*C. sakazakii*
29	SRL-65	Spice powder	India	Spice	TG	+	+	+	4	JQ674747, This report (Identical)	*C. sakazakii*
30	SRL-77	Spice powder	India	Spice	TG	+	+	+	1	CP011047, This report (Identical)	*C. sakazakii*
31	SRL-81	Spice powder	Pakistan	Spice	TG	+	+	+	7	JQ674750, This report (2-point-mutation)	*C. sakazakii*
32	SRL-82	Spice powder	India	Spice	TG	+	+	+	3	HQ646169, This report (Identical)	*C. sakazakii*
33	SRL-102	Ogbono seed	Ghana	Spice	TG	+	+	+	NOA	NOA	NOA
34	SRL-104	Spice powder	Jamaica	Spice	TG	+	+	+	1	CP011047, This report (Identical)	*C. sakazakii*
35	SRL-109	Paprika powder	Peru	Spice	TG	+	+	+	2	EU076546, This report (Identical)	*C. sakazakii*
36	SRL-124	Spice powder	Germany	Spice	TG	+	+	+	2	EU076546, This report (Identical)	*C. sakazakii*
37	SRL-126	Chili powder	Chile	Spice	TG	+	+	+	NOA	NOA	NOA
38	SRL-128	Spice powder	Kenya	Spice	TG	+	+	+	6	JQ674749, This report (identical)	*C. sakazakii*
39	SRL-160	Spice powder	El Salvador	Spice	TG	+	+	+	3	HQ646169, This report (Identical)	*C. sakazakii*
40	SRL-171	Spice powder	India	Spice	TG	+	+	+	1	CP011047, This report (Identical)	*C. sakazakii*
41	SRL-172	Spice powder	India	Spice	TG	+	+	+	6	JQ674749, This report (Identical)	*C. sakazakii*
42	SRL-179	Spice powder	India	Spice	TG	+	+	+	NOA	NOA	NOA
43	SRL-180	Spice powder	India	Spice	TG	+	+	+	6	JQ674749, This report (Identical)	*C. sakazakii*
44	SRL-181	Spice powder	India	Spice	TG	+	+	+	2	EU076546, This report (Identical)	*C. sakazakii*
45	SRL-187	Spice powder	India	Spice	TG	+	+	+	2	EU076546, This report (Identical)	*C. sakazakii*
46	SRL-51	Ready-to-eat snack	India	Snack	TG	+	+	+	4	JQ674747, This report (Identical)	*C. sakazakii*
47	SRL-72	Ready-to-eat snack	Vietnam	Snack	TG	+	+	+	2	EU076546, This report (Identical)	*C. sakazakii*
48	SRL-79	Ready-to-eat snack	India	Snack	TG	+	+	+	2	EU076546, This report (Identical)	*C. sakazakii*
49	SRL-99	Ready-to-eat snack	USA	Snack	TG	+	+	+	1	CP000783, This report (Identical)	*C. sakazakii*
50	SRL-131	Ready-to-eat snack	India	Snack	TG	+	+	+	6	JQ674749, This report (Identical)	*C. sakazakii*
51	SRL-152	Ready-to-eat snack	India	Snack	TG	+	+	+	1	CP011047, This report (Identical)	*C. sakazakii*

TG: growth of typical *Cronobacter* colonies observed on culture plates; * seven sets of primer were used to amplify the O-antigen 1–7 serotypes; NOA: no O-antigen PCR amplification; +: PCR positive. QPCR: quantitative polymerase chain reaction, also known as real-time polymerase chain reaction (Real-Time PCR).

**Table 3:** Published primers used in the study.

Target	Primer Name	Primer Sequence (5′–3′)	Reference
* 16S rRNA	616V	AGAGTTGATYMTGGCTC	[21]
	630R	CAKAAAGGAGGTGATCC	
* *rpo*B	*rpo*B-F	AACCAGTTCCGCGTTGGCCTGG	[22]
	*rpo*B-R	CCTGAACAACACGCTCGGA	
** *Wzy*, Serotype O1	wl-35646	CCCGCTTGTATGGATGTT	[23]
	wl-35647	CTTTGGGAGCGTTAGGTT	
** *Wzy*, Serotype O2	wl-37256	ATTGTTTGCGATGGTGAG	[23]
	wl-37257	AAAACAATCCAGCAGCAA	
** *Wzy*, Serotype O3	wl-37258	CTCTGTTACTCTCCATAGTGTTC	[23]
	wl-37259	GATTAGACCACCATAGCCA	
** *Wzy*, Serotype O4	wl-39105	ACTATGGTTTGGCTATACTCCT	[23]
	wl-39106	ATTCATATCCTGCGTGGC	
** *Wzy*, Serotype O5	wl-39873	GATGATTTTGTAAGCGGTCT	[23]
	wl-39874	ACCTACTGGCATAGAGGATAA	
** *Wzy*, Serotype O6	wl-40041	ATGGTGAAGGGAACGACT	[23]
	wl-40042	ATCCCCGTGCTATGAGAC	
** *Wzy*, Serotype O7	wl-40039	CATTTCCAGATTATTACCTTTC	[23]
	wl-40040	ACACTGGCGATTCTACCC	

* Generic primers, ** *Cronobacter sakazakii* O-antigen serotype specific primers.

**Table 4:** Species identification based on 16S rRNA and *rpo*B sequencing for *Cronobacter* isolates that failed to amplify using O-antigen (1–7) primer sets.

Sample Number	Description of Food Products	Country of Origin	16S rRNA Reference * (Sequence Variation, % Similarity)	16S rRNA Sequence Type	*rpo*B Reference ** (Sequence Variation, % Similarity)	*rpo*B Sequence Type
SRL-91	Pet food	Canada	GU122174, This report (Identical, 100%)	*C. malonaticus*	CP013940, This report (8-point-mutation, 99%)	*C. malonaticus*
			NR_102802, This report (Identical, 100%)	*C. turicensis*		
SRL-186	Pet food	USA	KF360293, This report (Identical, 100%)	*C. malonaticus*	CP013940, This report (Identical, 100%)	*C. malonaticus*
			KU364482, This report (Identical, 100%)	*C. sakazakii*		
SRL-12	Produce	Mexico	CP004091, This report (Identical, 100%)	*C. sakazakii*	CP013940, This report (3-point-mutation, 99%)	*C. malonaticus*
					JF330141, This report (3-point-mutation, 99%)	*C. sakazakii*
SRL-13	Produce	USA	CP012266, This report (Identical, 100%)	*C. dublinensis*	AB980795, This report (9-point-mutation, 98%)	*C. dublinensis*
			KU364468, This report (Identical, 100%)	*C. sakazakii*		
SRL-42	Produce	Colombia	KC818225, This report (2-point-mutation, 99%)	*C. malonaticus*	CP013940, This report (Identical, 100%)	*C. malonaticus*
			KU543632, This report (2-point-mutation, 99%)	*C. sakazakii*		
SRL-94	Produce	USA	KC109002, This report (2-point-mutation, 99%)	*C. malonaticus*	CP013940, This report (Identical, 100%)	*C. malonaticus*
			CP004091, This report (2-point-mutation, 99%)	*C. sakazakii*		
			HQ880409, This report (2-point-mutation, 99%)	*C. turicensis*		
SRL-102	Spice	Ghana	KC109002, This report (2-point-mutation, 99%)	*C. malonaticus*	JX425275, This report (3-point-mutation, 99%)	*C. sakazakii*
			CP004091, This report (2-point-mutation, 99%)	*C. sakazakii*		
			HQ880409, This report (2-point-mutation, 99%)	*C. turicensis*		
SRL-126	Spice	Chile	CP012266, This report (Identical, 100%)	*C. dublinensis*	JX425283, This report (6-point-mutation, 99%)	*C. dublinensis*
			KU364468, This report (Identical, 100%)	*C. sakazakii*		
SRL-179	Spice	India	KU364464, This report (Identical, 100%)	*C. sakazakii*	JF330150, This report (Identical, 100%)	*C. sakazakii*
